# Photodynamic Therapy of the Murine LM3 Tumor Using Meso-Tetra (4-N,N,N-Trimethylanilinium) Porphine

**Published:** 2007-12

**Authors:** L. L. Colombo, A. Juarranz, M. Cañete, A. Villanueva, J. C. Stockert

**Affiliations:** 1 *Institute of Oncology “A.H. Roffo”, University of Buenos Aires, Argentina;*; 2 *Department of Biology, Faculty of Sciences, Autonomous University of Madrid, Spain;*; 3 *Department of Biology, Faculty of Sciences, Autonomous University of Madrid, Spain;*; 4 *Department of Biology, Faculty of Sciences, Autonomous University of Madrid, Spain;*; 5 *Department of Biology, Faculty of Sciences, Autonomous University of Madrid, and Center for Biological Research, High Council for Scientific Research, Madrid, Spain*

**Keywords:** photodynamic therapy, photosensitizing drugs, cationic porphyrins, mammary adenocarcinoma, metastasis

## Abstract

Photodynamic therapy (PDT) of cancer is based on the cytotoxicity induced by a photosensitizer in the presence of oxygen and visible light, resulting in cell death and tumor regression. This work describes the response of the murine LM3 tumor to PDT using meso-tetra (4-N,N,N-trimethylanilinium) porphine (TMAP). BALB/c mice with intradermal LM3 tumors were subjected to intravenous injection of TMAP (4 mg/kg) followed 24 h later by blue-red light irradiation (λ_max_: 419, 457, 650 nm) for 60 min (total dose: 290 J/cm^2^) on depilated and glycerol-covered skin over the tumor of anesthetized animals. Control (drug alone, light alone) and PDT treatments (drug + light) were performed once and repeated 48 h later. No significant differences were found between untreated tumors and tumors only treated with TMAP or light. PDT-treated tumors showed almost total but transitory tumor regression (from 3 mm to less than 1 mm) in 8/9 animals, whereas no regression was found in 1/9. PDT response was heterogeneous and each tumor showed different regression and growth delay. The survival of PDT-treated animals was significantly higher than that of TMAP and light controls, showing a lower number of lung metastasis but increased tumor-draining lymph node metastasis. Repeated treatment and reduction of tissue light scattering by glycerol could be useful approaches in studies on PDT of cancer.

## INTRODUCTION

Photodynamic therapy (PDT) of cancer is based on cell damage induced by a photosensitizer (PS) in the presence of visible light and molecular oxygen. The process generates reactive oxygen species which cause destruction of neoplastic cells and represents a promising therapy for pulmonary, gastrointestinal, urinary and skin tumors (1-4). Localization of PSs in cell organelles, photodynamic mechanisms and signaling pathways of apoptotic cell death are now important issues related to PDT research (5-7). In order to improve the results obtained with the original hematoporphyrin derivatives, 2nd-generation PSs are now being evaluated. Examples include dyes, synthetic porphyrins, phthalocyanines and porphycenes (5, 7, 8-10).

**Figure 1:**
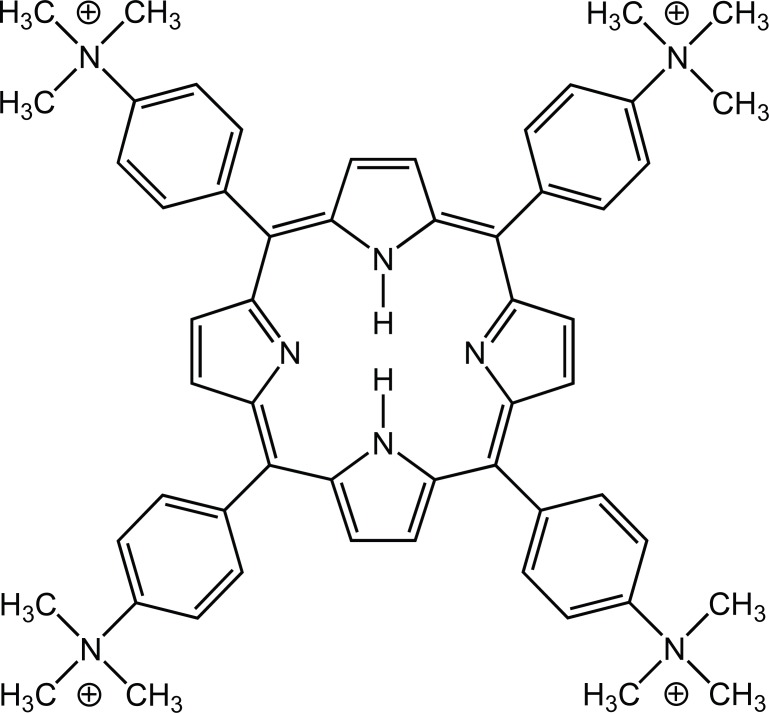
Chemical structure of TMAP.

The cationic porphyrin meso-tetra (4-N,N,N-trimethylanilinium) porphine (TMAP) (Fig. 1) is a PS with a high quantum yield of ^1^O_2_ formation (Φ_Δ_=0.77) (11). TMAP is an outside DNA binder (12-14), inserts into branched DNA structures causing photodamage (15), and induces photodynamic inactivation of bacteria (16). Photodynamic treatments of cell cultures (17-19) and tumor models (20-22) using several cationic porphyrins are known, but as no reports on TMAP photosensitization of tumors have been published, we describe here some preliminary results of repeated TMAP-PDT using the murine LM3 tumor.

## MATERIAL AND METHODS

Inbred female BALB/c mice, 2-4 months old, were obtained from the Breeding Area of the Institute of Oncology and kept in a temperature- and light-controlled room with free access to water and dietary chow. Animal care was provided in full compliance with regulations for protection of animals. LM3 cells (from a BALB/c-transplantable mammary adenocarcinoma with moderate metastatic ability) (23) were grown at 37°C in plastic flasks (Falcon) in a humidified 5% CO_2_ atmosphere, using minimum essential medium (MEM, Gibco) with 5% fetal bovine serum, 2 mM L-glutamine, and 80 μg/ml gentamycin. For inoculation into mice, cells were harvested from subconfluent cultures with trypsin-EDTA, washed thoroughly with MEM, and resuspended in the same medium. LM3 tumors were produced by intradermal inoculation of 3 × 10^5^ cells in 0.05 ml MEM in the depilated left flank of non-anesthetized animals.

LM3-bearing mice were treated with intravenous injections of meso-tetra(4-N,N,N-trimethylanilinium) porphine tetra-p-tosylate salt (TMAP, Aldrich; molecular weight: 1866) (4 mg/kg; 100 μg in 0.5 ml of 0.9% NaCl for each animal of 25 g weight, in agreement with the work of Villanueva and Jori (20) using the similar porphyrin, meso-tetra(4-N-methylpyridinium) porphine tetra-p-tosylate salt (TMPyP; molecular weight: 1363). Comparatively, TMAP dosage (2.2 μmoles/Kg) was somewhat lower than that of TMPyP (2.9 μmoles/Kg). 24 h after TMAP treatment, mice were anaesthetized with an intraperitoneal injection of 0.01 ml/g body weight Ketalar (Parke Davis, 0.23 mg/ml) and Rompum (Bayer, 0.14 mg/ml) cocktail. The tumor area was irradiated with blue-red light (290 J/cm^2^, 80 mW/cm^2^ for 60 min) from a photopolymerizing device for odontological use, equipped with a 35 W-12 V halogen lamp (KO 13165, Philips), an ellipsoidal dichroic reflector, a combination of blue and red filters (transmission peaks at 419, 457, and 650 nm), and a heat absorbing filter (KG-1 glass, Schott) as previously described (22). The light intensity was measured with a Broadband Power Energymeter 13 PEM 001 (Melles Griot Laser Optics). The light beam was guided by internal reflection through a glass rod (length: 10 cm, diameter: 1 cm) whose tip was placed directly on the depilated skin covering the tumor area. To reduce light scattering, skin was soaked with glycerol (22), with a thin layer of glycerol always remaining between skin and the glass rod tip during irradiation.

One or two weeks after LM3 cell inoculation, mice with tumors showing an average diameter (AD) of about 3 mm were subjected to control and PDT treatments: (a) tumors from 4 animals were left untreated, (b) tumors from 4 animals were irradiated without previous TMAP injection, (c) tumors from 7 animals were injected with TMAP but non irradiated, and (d) tumors from 9 animals were subjected to PDT with TMAP + light. All control and PDT treatments were applied on the depilated and glycerol-covered skin over the tumor of anaesthetized mice and repeated two times with an interval of 48 h. Tumor ADs were assessed using a Vernier caliper and the equation AD = ^3^√x.y.z, where x, y, and z are the orthogonal diameters of tumors. At death, the tumor size as well as the number of lung and tumor-draining lymph nodes metastasis was evaluated under a Wild 3 stereomicroscope. Statistic analysis was performed with the Graph Pad Stat program.

## RESULTS

**Figure 2:**
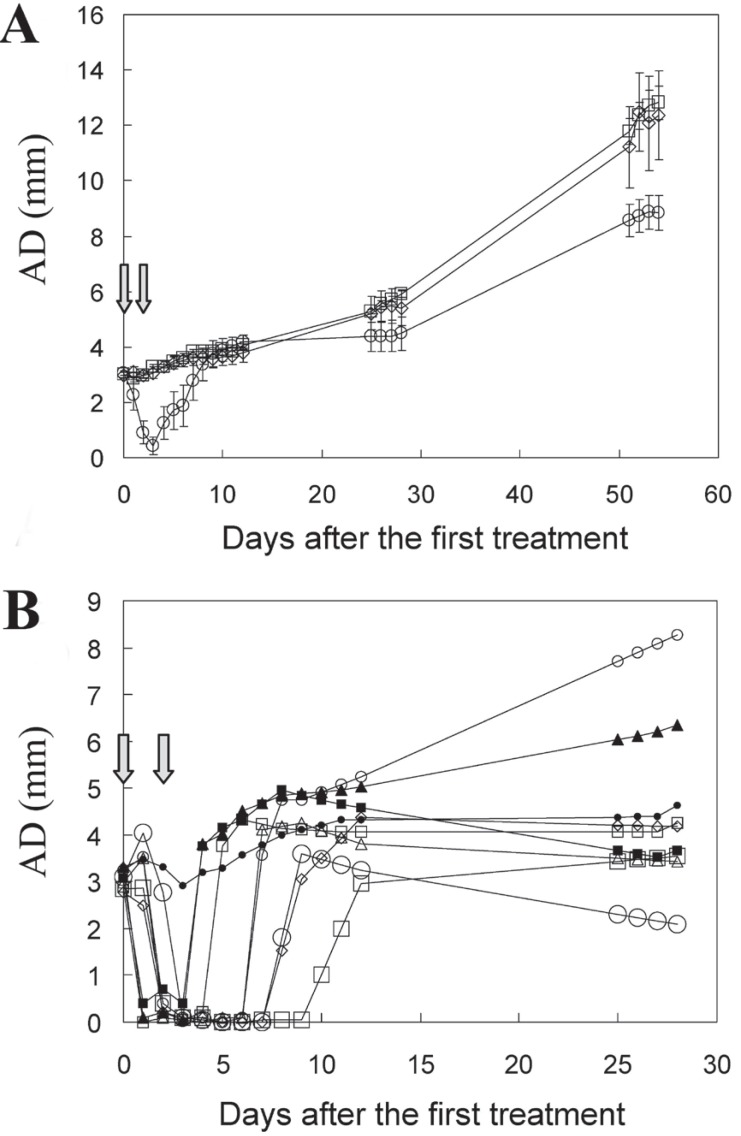
A, growth kinetics of LM3 tumors treated 2 times (arrows) with light alone (squares), TMAP alone (diamonds), or TMAP + light (circles); B, expansion of the growth curve of individual tumors subjected to PDT. Mean AD values ± SEM bars are shown.

No significant differences were found in the tumor growth of the three control groups. Tumors treated two times with TMAP or with light alone showed the same growth rates as untreated controls, thus ruling out any contribution of drug and light separately to PDT response. Preliminary studies using a single PDT treatment showed a very poor response (not shown). In contrast, after two PDT rounds (TMAP + light), tumors showed a clear regression and delay in the growth rate (Fig. 2A).

Although the general response of LM3 tumors was (a) reduction of tumor size and (b) growth delay when relapses occurred, considerable heterogeneity was observed in the individual response to PDT. Almost complete but transitory tumor regression (AD from 3 mm to less than 1 mm) occurred in 8/9 PDT-treated animals [days, median (range): 5 (3-8)], whereas no tumor regression was found in 1/9. To compare the response of each animal to PDT, the growth of individual tumors is shown in Fig. 2B.

**Figure 3:**
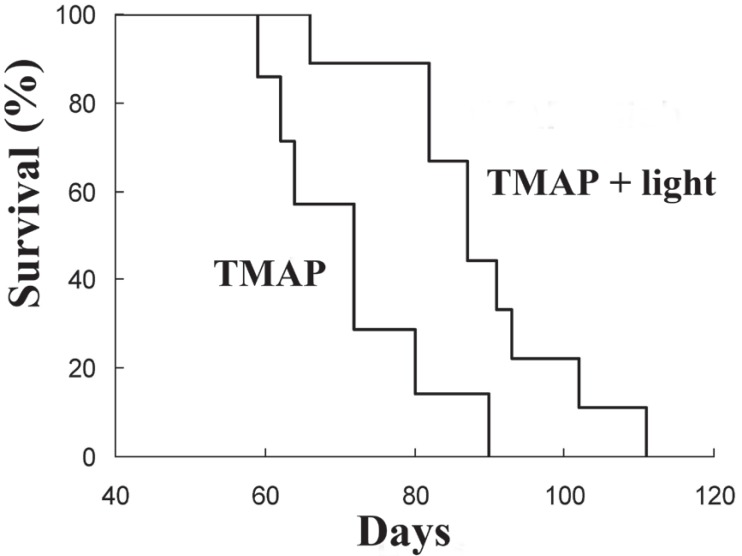
Survival of tumor-bearing animals after treatments with TMAP alone and TMAP + light.

**Figure 4:**
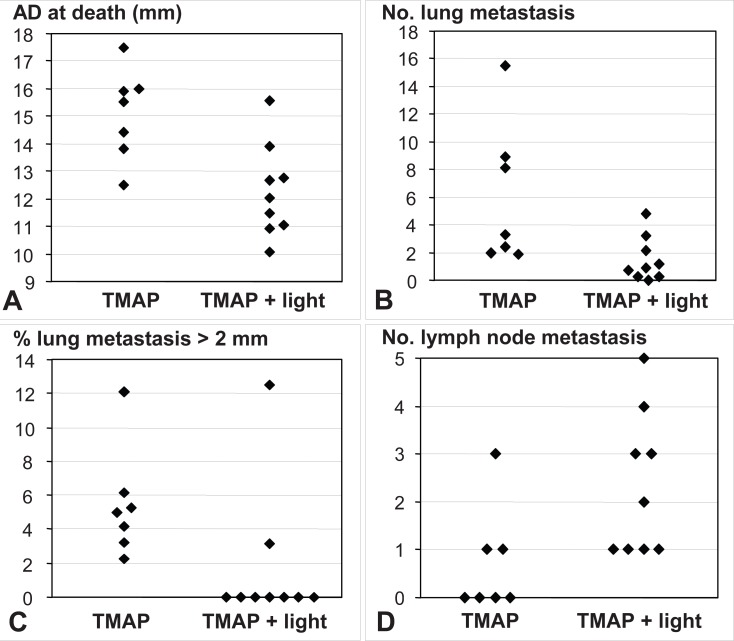
Size and metastasis parameters of control (TMAP alone) and PDT-treated tumors. A, average diameter (AD) of tumors at death (*p*=0.0115; *p*<0.05); B, number of lung metastasis (*p*=0.0164; *p*<0.05); C, percent of lung metastasis greater than 2 mm (*p*=0.0205; *p*<0.05); D, number of tumor-draining lymph node metastasis (*p*=0.0252; *p*<0.05).

A significant increase in the survival of PDT-treated animals was observed when compared to drug-alone controls (Fig. 3). The survival of PDT vs. control animals [days, median (range)] was 87 (66-111) vs. 72 (59-90), *p*=0.015 (Mann-Whitney test, *p*<0.05). The higher survival of PDT group over controls did not correspond simply to the regression gap (15 days vs. 5 days in average, respectively), but it represented more than this time period. The tumor size of PDT group at death also was smaller than that of controls (Fig. 4A). PDT-treated mice showed a number of lung metastasis lower than drug-alone controls [median (range): 9 (0-48) vs. 33 (19-155)] (Fig. 4B), and with smaller size [percent of metastasis greater than 2 mm, mean (range): 0 (0-12.5) vs. 5 (2.2-12.1)] (Fig. 4C). However, PDT-treated animals presented a greater amount of lymph node metastasis than drug-alone controls (Fig. 4D). This unexpected and intriguing feature deserves further investigation.

## DISCUSSION

PDT responses of animal tumors to different PSs and protocols show considerable variability, results ranging from partial regression or delay in the tumor growth (8, 20, 21, 24-26), to prolonged and in some cases complete tumor regression (22, 27-29). It is very frequent that after temporary remission, tumors subjected to PDT start to grow again (20, 30). Present results showing an almost complete but temporary regression as well as growth delay of PDT-treated LM3 tumors are in agreement with most of these observations, and confirm the heterogeneous response of experimental tumors to PDT.

It is known that TMAP photoinactivates bacteria (16), but PDT applications for this PS seem to have been overlooked. In the present work, the following methodological features were applied: (a) intradermal tumors quite accessible to light, (b) reduction of light scattering of the skin by glycerol soaking, and (c) repeated PDT treatment. However, the response was rather modest, and after almost complete regression of most tumors, they started to growth again with time. Relapses likely originated from tumor cells that were not inactivated by PDT, which could be due to variations in the penetration of light within the tumor. Interestingly, when used at somewhat lower dosage than a similar porphyrin (TMPyP), TMAP induced more prolonged regression and increased survival, whereas TMPyP only caused a very small and transitory delay in tumor growth (20).

Different PDT effects on lung and lymph node metastasis were found. A decrease in both the number and size of lung metastasis was observed, whereas an opposite effect occurred in lymph node metastasis. This intriguing fact opens the question of why the metastasis from a PDT-treated tumor can show different behavior when they are growing within different organs. The study of these differences could open an interesting field of research.

Possible clinical implications from the present results include: (a) PDT-treated mice showed higher survivals than the duration of tumor regression (15 days vs. 5 days, respectively), and (b) the growth rate of tumor relapses was slower than control tumors, which is consistent with the increased survival. These features are important for oncological applications. Taking into account that the life span of mice is about 2 years, one week in the life of a mouse would be equivalent to 10 months in human. Although far from complete, the results presented here indicate that repeated treatments with TMAP and reduction of tissue light scattering could represent useful strategies for PDT studies in experimental and clinical oncology.
